# Manganese Uptake and Lethality in the Sea Star *Asterias rubens:* Effect of Hypoxia

**DOI:** 10.1007/s00128-025-04037-6

**Published:** 2025-04-12

**Authors:** Poul Bjerregaard, Søren Nordahl Hansen

**Affiliations:** 1 Ecotoxicology Group, Department of Biology, University of Southern Denmark, Campusvej 55, 5230 Odense M, Denmark; 2 Danish Appeals Boards Authority, Ministry of Industry, Business, and Financial Affairs, Copenhagen, Denmark

**Keywords:** Manganese, Hypoxia, Sea star, Accumulation, Toxicity

## Abstract

Oxygen depletion due to eutrophication in marine waters has been an increasing problem worldwide and during oxygen depletion events, benthic organisms may concurrently be exposed to hypoxia and manganese leaching from reduced sediments. In this investigation the uptake and toxic effects of manganese under normoxia and hypoxia were studied in the sea star *Asterias rubens*. Exposure to 1 mg Mn L^−1^ for 4 weeks resulted in increased manganese concentrations in tube feet and pyloric caeca, whereas the concentrations in the aboral body wall showed no statistically significant changes. Manganese concentrations and accumulation did not differ between hypoxia (25% oxygen saturation) and normoxia; mortality was not observed in either of these exposure scenarios. Exposure to 0, 25, 35 and 50 mg Mn L^−1^ resulted in a time and concentration dependent increase in mortality over 7 d, aggravated by hypoxic conditions. Hypoxic conditions significantly reduced average survival time at exposure to 50 mg Mn L^−1^.

## Introduction

Although manganese is an essential metal in living organisms both as a constituent in and important activator for several enzymes (i.e. Zelko et al. 2002), exposure to elevated concentrations may lead to toxic manifestations in aquatic organisms (e.g. Hansen and Bjerregaard 1995).

Oxygen depletion due to eutrophication in marine waters has been an increasing problem worldwide (reviewed by Diaz and Rosenberg 2008) and more specifically in some north European coastal areas such as the Swedish west coast (Rosenberg et al. 1990), parts of the Baltic and the Danish belt area (Hansen 2021). Porewater concentrations of manganese in anoxic marine sediments of 13 mg Mn^++^ L^−1^ (Thamdrup et al. 1994) and 19 mg Mn^++^ L^−1^ (Magnusson et al. 1996) have been reported. Under hypoxic and anoxic conditions in bottom waters, the flux of reduced manganese (Mn^++^) from the sediment pore water increases and concentrations in the order of 1 mg Mn^++^ L^−1^ (Kremling 1983) of dissolved manganese in the water column may be reached; background manganese concentrations in oxic, marine waters generally are in the 0.01–0.05 μg L^−1^ range (Kremling 1983; Landing et al. 1995). Manganese liberated as Mn^++^ from the sediment during hypoxic conditions is re-oxidized to MnO_2_ fairly slowly (in the order of weeks) after reintroduction of oxygen to the water (Dehairs et al. 1989). Therefore, benthic organisms may encounter concurrent exposure to both increased manganese concentrations and hypoxic conditions.

Manganese has the potential to accumulate in aquatic organisms from water (Baden and Eriksson 2006; Baden et al. 1995, 1999, 2003; Hansen and Bjerregaard 1995; Rouleau et al. 1995) and food (Hansen and Bjerregaard 1995) and adverse effects of the accumulated manganese have been observed in several benthic invertebrates (Fedyunin et al. 2019; Hansen and Bjerregaard 1995; Oweson et al. 2006, 2008, 2010; Skold et al. 2015).

Starfish are important predators in many benthic ecosystems and the common starfish *A. rubens* may occur in high numbers in populations of blue mussels *Mytilus edulis/trossulus* (e.g. Anger et al. 1977; Dolmer 1998; Khaitov et al. 2018; Lowen et al. 2013; Norberg and Tedengren 1995; Reimer et al. 1995). *A. rubens* itself is preyed upon by fish and birds in coastal areas; e.g. by the herring gull *Larus argentatus* (Bukacinska et al. 1996). Contaminants in sea stars may also find their way into human food items because sea stars are harvested locally to be used as a protein source in the breeding of pigs (Skovborg 2018) and poultry (Afrose et al. 2016).

The combined effects of exposure to hypoxia and manganese have only been sparsely investigated (Baden et al. 1995) and since only few investigations have been carried out on the kinetics of manganese in benthic organisms, the purpose of the present study was to investigate the interaction between hypoxia and manganese in the sea star *A. rubens*.

## Materials and Methods

### Experimental Animals

Sea stars *Asterias rubens* were collected by hand at water depths less than 1 m in Little Belt, Denmark (55° 29′ 39ʺ N; 9°42′7ʺ E). This is an area in which animals normally experience fluctuating salinities– typically between 12 and 28‰; salinity and temperature on the day of collection were 17.4 ± 0.1‰ and 15.5 ± 0.5 °C.

All animals were acclimated in the laboratory for 7 days prior to experiments. They were not fed during the acclimation period.

### Exposure Procedures and General Laboratory Conditions

The temperature in the laboratory experiments was maintained at 14.5 ± 0.5 °C and 12 h light/dark period was used. Great Belt water was used in the experiments and salinity was determined by means of a conductivity meter (Struers CDM3). Water was changed twice a week. The water was aerated by means of air stones. No sediment was placed in the aquaria and the sea stars were not fed during experiments.

### Manganese Analyses

Approximately 100 mg freeze dried tissue were transferred to 2 mL concentrated HNO_3_ in 20 mL Pyrex glass tubes that were gradually heated to 120 °C. The acid was evaporated, and the samples dissolved in 2 mL 0.2% nitric acid. Concentrations of manganese were determined by means of a Perkin Elmer 4000 atomic absorption spectrometer. The quality of the determinations was validated by incorporation of a certified reference material from the Canadian National Research Council (TORT-1 standard; lobster hepatopancreas) with a certified value of 23.4 ± 1.0 μg Mn g^−1^ in each series. The average recovery was 19.3 ± 1.2 μg Mn g^−1^; the values presented are not corrected for recovery.

### Hypoxia

Hypoxia was obtained by mixing N_2_ (AGA) and atmospheric air (AGA) from 50 L gas cylinders by means of a GM-602 gas mixer (ADC). The gas mixture was admitted to the aquaria via bubbling stones. The aquaria were covered and closed with lids. The oxygen concentrations were controlled daily by means of a PHM73 oxygen meter (Radiometer). The nominal oxygen concentration in the hypoxic group was 25% oxygen saturation versus 100% in the control.

### Chemicals

Concentrated nitric acid and manganese MnC1_2_·4H_2_0 were supplied by Merck (all pro analysis).

## Experiments

### Effect of Hypoxia Uptake on Mn^++^ Uptake

Accumulation of manganese from 1.0 mg Mn^++^ L^−1^ during experimental hypoxia and normoxia was studied in 11 groups of 6 sea stars (body wet wt 9 ± 5 g) held in 10 L polystyrene aquaria. Three of the aquaria served as control, two were exposed only to hypoxia, three only to manganese (1.0 mg Mn^++^ L^−1^) and three to both hypoxia and manganese. The aquaria exposed to hypoxia had an oxygen saturation on 25 ± 1%. Salinity of the water was 18 ± 3‰. The experiment was initiated in Mid-August. After 1-, 2- and 4-weeks’ exposure, 6 animals from each of the manganese exposed groups were sacrificed. Groups not exposed to manganese were sampled day 0 and 2 and 4 weeks into the experiment. Samples of pyloric caeca, tube feet and aboral skin were dissected out. The dissected tissues were blotted dry with paper towels, weighed, and frozen at  − 18 °C. The frozen samples were freeze dried (Hetosicc freeze dryer) for at least 3 days and the dry weight was recorded. Unfortunately, some of the samples were lost due to equipment failure.

### Effect of Hypoxia and Mn^++^ on Survival

The toxicity of Mn^++^ and hypoxia towards sea stars (body wet wt 9 ± 3 g) was examined by exposing 8 groups of 10 sea stars in 10-L polystyrene aquaria to 0, 25, 35 and 50 mg Mn^++^ L^−1^ with two groups at each concentration. At each of these concentrations, one group exposed at 26 ± 2% oxygen saturation and the remaining group at 100% oxygen saturation. Salinity of the water was 19 ± 2‰. The mortality was registered every 24 h and dead sea stars were removed from the aquaria. The experiment was carried out in Mid-July.

### Data Handling and Statistical Treatment

During the uptake phase, data for the concentration of manganese were fitted to either linear ([Mn] = a + b*days) or polynomial ([Mn] = a + b*days + c*days^2^) equations. Curve fitting was carried out in the programme FigP. One or two-way analysis of variance (ANOVA) were used in statistical evaluation of the data (SYSTAT, version 13). 0.05 was used as level of significance.

## Results

### Effect on Mn Accumulation of Combined Exposure to Hypoxia and Mn

**Fig. 1:**
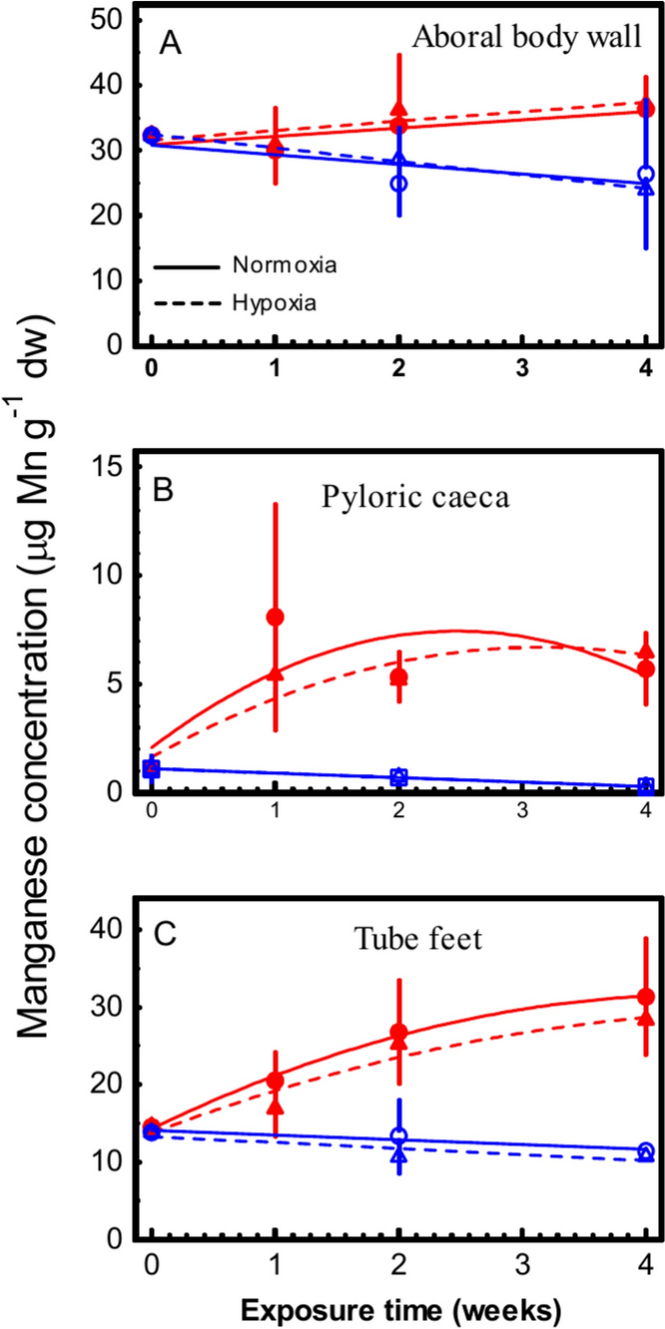
*Asterias rubens.* Concentrations of manganese in aboral body wall (**A**), pyloric caeca (**B**) and tube feet (**C**) of sea stars exposed to 1 mg Mn-MnCl_2_ L^−1^ at 100% (red filled Circle; solid lines) or 25 ± 1% (Red filled triangle; broken lines) oxygen saturation. Control animals at 100% (Open circle; solid lines) or 25 ± 1% (Open triangle; broken lines) oxygen saturation had no manganese added. Mean ± SEM for n = 1 − 6

Aboral body wall, pyloric caeca and tube feet had manganese concentrations in unexposed animals of 31–32, 1–2 and 13–14 µg Mn g^−1^ dry weight (Fig. 1). The normoxia control group appeared to lose some manganese from the tissues during the 4 weeks exposure, but the decreases in manganese concentrations were not statistically significant (Table 1). During exposure to 1.0 mg Mn^++^ L^−1^ under normoxia, the tube feet accumulated manganese to significant levels (Fig. 1C; Table 1), whereas changes in the aboral body wall and pyloric caeca were not statistically significant (Fig. 1A and B, Table 1).

**Table 1:** Equations for the regression lines shown in Fig. 1

Tissue		a	b	c	*p*
Aboral body wall	Control + normoxia	30.8	− 1.47		Not sig
	Control + hypoxia	32.5	− 2.07		Not sig
	Mn exposed + normoxia	30.9	1.27		Not sig
	Mn exposed + hypoxia	31.6	1.44		Not sig
Pyloric caeca	Control + normoxia	1.1	− 0.21		Not sig
	Control + hypoxia	1.1	− 0.21		Not sig
	Mn exposed + normoxia	2.08	4.34	− 0.88	Not sig
	Mn exposed + hypoxia	1.64	3.2	− 0.51	*p* < 0.05
Tube feet	Control + normoxia	14.1	− 0.60		Not sig
	Control + hypoxia	13.3	− 0.77		Not sig
	Mn exposed + normoxia	14.3	7.73	− 0.86	*p* < 0.01
	Mn exposed + hypoxia	13.7	6.05	− 0.58	*p* < 0.01

[Mn] = a + b*days + c*days^2^

Exposure to hypoxia did not affect manganese concentrations compared to the normoxic conditions, neither in the manganese exposed nor in the control animals (Fig. 1). Under hypoxia and manganese exposure, pyloric caeca accumulated manganese to increased levels (Fig. 1b, Table 1).

### Effect on Survival of Combined Exposure to Hypoxia and Mn

**Fig. 2:**
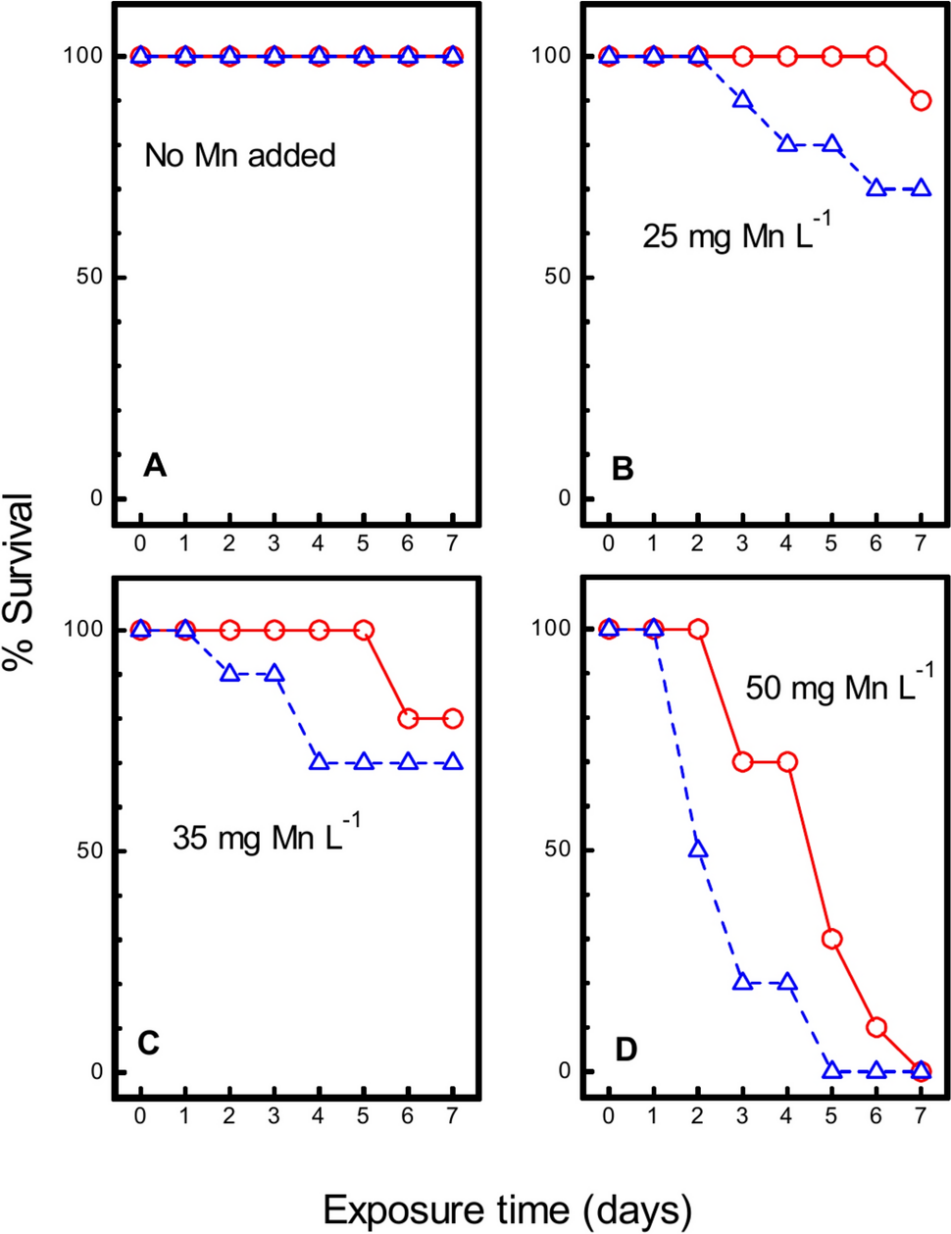
*Asterias rubens*. Survival of sea stars exposed to 0 (**A**), 25 (**B**) 35 (**C**) or 50 (**D**) mg Mn-MnCl_2_ L^−1^ at 100% (open red circle; solid lines) or 26 ± 2% (Open triangle; broken lines) oxygen saturation. 10 animals in each group

No mortality was registered in the control group exposed to neither Mn nor hypoxia (Fig. 2A). At exposure to 25 mg Mn L^−1^ alone, mortality was not registered until day 7 (Fig. 2B). At exposure to 35 mg Mn L^−1^ alone, mortality set in at day 6 (Fig. 2C). At 50 mg Mn L^−1^, mortality set in at day 3 and all animals were dead at day 7 (Fig. 2D).

**Fig. 3:**
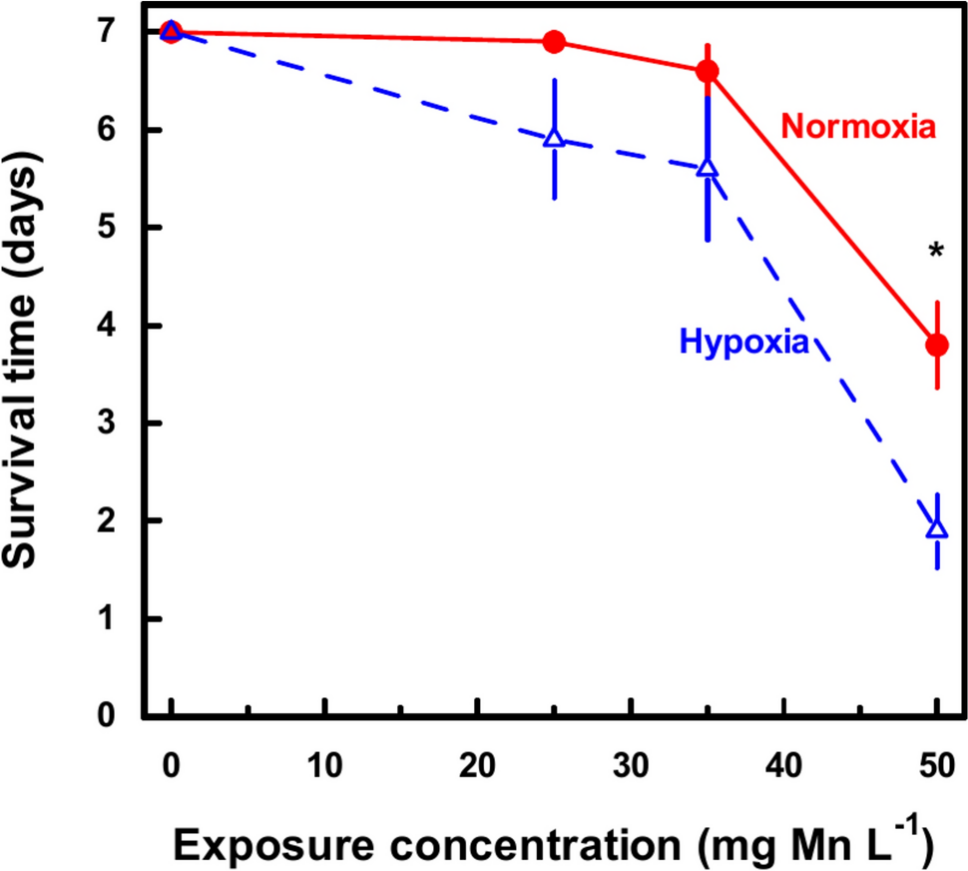
*Asterias rubens*. Average survival times for sea stars exposed to exposed to 0, 25, 35 or 50 mg Mn L^−1^ at 100% (open red circle; solid lines) or 26 ± 2% (Open triangle; broken lines) oxygen saturation Mean ± SEM for 10 sea stars shown. *p < 0.05

Concurrent exposure to hypoxia and manganese aggravated the mortality in the groups exposed to 25, 35 and 50 mg Mn L^−1^ relative to the mortality in the groups exposed to manganese alone (Fig. 2B–D). The average survival time was significantly lower in the group concurrently exposed to hypoxia and 50 mg Mn L^−1^ compared to the group exposed to 50 mg Mn L^−1^ alone (Fig. 3).

## Discussion

No mortality was registered in *Asterias rubens* exposed to hypoxic condition in the present investigation, neither for sea stars exposed to 26 ± 2% oxygen saturation at 19 ± 2‰ for 7 d, nor for sea stars exposed for 4 weeks to an oxygen saturation of 25 ± 1% at a salinity of the water at 18 ± 3‰. The present results corroborate the observations that no mortality was registered in *A. rubens* exposed to 1.8 mg O_2_ L^−1^ (24% saturation) for 14 days a 7.5 °C in natural Atlantic Ocean seawater (Wahltinez et al. 2023). Also, no mortality was reported for *A. rubens* exposed to hypoxia at 14–16% air saturation for 3 d at 12 °C and 33‰ (Oweson et al. 2010). Medium survival time for *A. rubens* in almost anoxic (0.15 mg O_2_ L^−1^) 15‰ seawater was 84 h (Theede et al. 1969). The behaviour of benthic invertebrates begins to be affected at 2.8 mg O_2_ L^−1^, leading to escape responses in the form of increased vertical and horizontal movements (Riedel et al. 2008) and e.g. arm tipping in ophiuroids (Riedel et al. 2014). Hypoxia (< 2.8 mg O_2_ L^−1^) affects species-species interactions among benthic invertebrates (Riedel et al. 2014) and mortality generally occurs at moderate (2 mg O_2_ L^−1^) to severe (0.5 to 1 mg O_2_ L^−1^) hypoxia (Riedel et al. 2014); the latter may cause mortality after 1–2 d exposure (Riedel et al. 2014).

No mortality was reported for *A. rubens* exposed to 15 mg Mn L^−1^ for 3 d at 12 °C and 33‰ (Oweson et al. 2010). Sea stars exposed to 10 and 25 mg Mn L^−1^ for 7 days showed no mortality, while animals exposed to 50, 100 or 200 mg Mn L^−1^ at 18 ± 0.9‰ and 14 °C had median survival times at 14.4, 18 and 72, hours, respectively (Hansen and Bjerregaard 1995). The survival time for sea stars exposed to 50 mg Mn L^−1^ was longer in the present investigation (112 h) than reported by (Hansen and Bjerregaard 1995); animals were larger in the present experiment (9 ± 3 g) as opposed to 5 ± 2 g reported by Hansen and Bjerregaard (1995) and salinity was also slightly higher in the present experiment which was carried out in July as opposed to September: It is unknown if these differences explain the longer survival time in the present experiment.

No mortality was reported for *A. rubens* exposed to 15 mg Mn L^−1^ and hypoxia at 14–16% air saturation for 3 d at 12 °C and 33‰ (Oweson et al. 2010) and these results are in accordance with the results of the present investigation, in which mortality after 3 d exposure at 26 ± 2% oxygen saturation only starts to set in at manganese concentrations of 25 and 35 mg Mn L^−1^ and concurrent exposure to 1 mg Mn L^−1^ and 25 ± 1% oxygen saturation for 4 weeks does not lead to any mortality.

Concurrent exposure to mild hypoxia does aggravate the effects of exposure to fairly high concentrations of manganese in the sea water, but it is questionable if this effect would ever be exerted under field conditions. Porewater concentrations of manganese in anoxic marine sediments of 13 mg Mn^++^ L^−1^ (Thamdrup et al. 1994) and 19 mg Mn^++^ L^−1^ (Magnusson et al. 1996) have been reported and under hypoxic and anoxic conditions in bottom waters, the flux of reduced manganese (Mn^++^) from the sediment pore water increases and concentrations in the order of 1 mg Mn^++^ L^−1^ (Kremling 1983) of dissolved manganese in the water column may be reached; still, this concentration of manganese in the water column does not appear to be detrimental to the sea stars.

Exposure to hypoxia did not affect the accumulation of manganese in the tissues of *A. rubens* from 1 mg Mn^++^ L^−1^. In the shore crab *Carcinus maenas*, hypoxia moderately affected the accumulation of manganese from seawater, resulting in slightly higher concentrations in the midgut gland and slightly lower concentrations in the exoskeleton, the tissue in which the vast majority of the body burden of manganese is found in Bjerregaard et al. (2024).

## Data Availability

Data sets are available upon contact to PB.
